# Objective screen time and smartphone addiction symptoms in defining risky smartphone use: A cross-sectional study of university students

**DOI:** 10.1007/s11845-026-04470-4

**Published:** 2026-06-01

**Authors:** Burcu Bakar Kahraman, Rümeysa Yeni Elbay

**Affiliations:** 1 Department of Psychiatry, Sultan II. Abdulhamid Han Training and Research Hospital, University of Health Sciences, Uskudar, Istanbul, Türkiye; 2 Department of Psychiatry, Faculty of Medicine, Istanbul Medeniyet University, Istanbul, Türkiye; 3 Göztepe Prof. Dr. Süleyman Yalçın City Hospital, Doktor Erkin Street, Kadıköy/Istanbul, Türkiye

**Keywords:** Smartphone, Problematic smartphone use, Mindfulness, Insight, Addiction, Screen time

## Abstract

**Background:**

Prolonged and problematic smartphone use is an important global concern, particularly among young adults.

**Aims:**

This study aimed to define a profile of risky smartphone use and to investigate the sociodemographic, environmental, and psychological factors associated with it.

**Methods:**

Data were collected using a sociodemographic and clinical data form, the Smartphone Addiction Scale-Short Version (SAS-SV), the Mindful Attention Awareness Scale (MAAS), and the Insight Scale (IS). Daily screen time was obtained from participants’ smartphone screen-time records. Risky use was defined as screen time ≥ 6 hours/day and/or an SAS-SV score above the cutoff.

**Results:**

A total of 297 university students were included. In bivariate comparisons based on both screen time and SAS-SV scores, no significant between-group differences were found in terms of sex, field of study, age at first smartphone ownership, or accuracy of estimated screen time. In contrast, the high SAS-SV group was characterized by a higher rate of dormitory living, whereas the low SAS-SV group more frequently lived with family (*p* = 0.020). In the multivariable model, age (p < 0.001) and total MAAS score (p < 0.001) independently and inversely predicted belonging to the risky use group. The prevalence of photo- and video-sharing applications was higher in the risky use group.

**Conclusions:**

Younger age and lower mindfulness seem to be the main determinants of risky smartphone use. Rather than independently predicting risk, insight may reflect how individuals perceive and report their behavior. Future studies should use holistic models incorporating duration, psychological characteristics, and contextual factors.

## Introduction

Approximately 7.5 billion people worldwide currently use smartphones, and this number is expected to reach 8 billion by 2028 [1]. Although smartphones have become indispensable because of the advantages they provide in communication, education, and access to information, growing evidence indicates that uncontrolled and excessive use may impair social functioning, academic performance, and psychological well-being. Accordingly, dysfunctional smartphone use has increasingly been recognized as an important public health issue.

There is no clear terminological consensus regarding the pathological dimension of smartphone use. In the literature, terms such as problematic use, maladaptive use, excessive use, harmful use, and addiction are often used interchangeably. This behavioral pattern also overlaps phenomenologically and neurobiologically with internet addiction, gaming disorder, and other behavioral addictions. Difficulties in emotion regulation, heightened impulsivity, compulsive behavioral patterns, and a range of psychiatric comorbidities are commonly observed in such behavioral addictions. Similarly, excessive smartphone use has been associated with various mental and physical health problems, including anxiety, depression, low self-esteem, and sleep disturbance [2–4]. However, some authors argue that the current evidence remains insufficient to classify smartphone use within the addiction framework [5].

One of the most frequently used indicators in studies evaluating smartphone use is screen time. However, in most studies that adopt screen time as an indicator, the data are based on participants’ subjective estimates. This increases the risk of recall bias and measurement error. Although objectively recorded screen-time data obtained directly from the device appear to be more reliable, they are not sufficient on their own to identify problematic use [6]. Problematic use is not defined solely by the amount of time spent on the device, but rather by multidimensional criteria such as loss of control, spending more time than intended, functional impairment, and persistence despite negative consequences. Indeed, some studies have reported only weak associations between screen time and problematic use [7]. The most commonly used tools for assessing problematic smartphone use are self-report scales; however, these instruments are also prone to response bias and may be influenced by the individual’s capacity to recognize and evaluate their own behavior. For this reason, in the present study, time-based intensive use and self-report-based problematic use were evaluated together, and a broader construct of risky use was defined to encompass both dimensions. In addition, the Insight Scale was used to examine the individual’s capacity to recognize and evaluate their own emotions, thoughts, and behaviors.

Previous research has identified numerous factors associated with increased risk of problematic smartphone use, including mental health problems, emotional factors, academic stress, peer victimization, and family-related variables [8]. In particular, there is substantial evidence indicating a significant negative association between mindfulness and problematic smartphone use [9, 10]. A similar finding has been reported for the association between screen time and mindfulness, although at a weaker level [11]. By contrast, the relationship between insight and patterns of smartphone use has not been investigated. Yet an individual’s capacity to recognize, evaluate, and critically reflect on their own behavior may be important both for interpreting self-report-based measures and for understanding patterns of dysfunctional use.

The aim of this study was to examine the psychological and environmental variables potentially associated with risky smartphone use in a cross-sectional sample of university students. The novel aspect of this study lies in its simultaneous evaluation of screen time and a self-report-based addiction scale for assessing smartphone use, while also considering mindfulness and insight together. This approach may contribute to the literature by clarifying the shared and distinct aspects of intensive and problematic smartphone use. We hypothesized that evaluating smartphone use through a broader risky use profile, rather than relying on a single indicator, would provide a stronger framework for identifying dysfunctional patterns of use.

## Materials and methods

A total of 297 university students who agreed to participate were included in this cross-sectional study. Inclusion criteria were being 18 years of age or older, being a university student, using a smartphone, and volunteering to participate. Exclusion criteria were having a history of severe psychiatric illness (intellectual disability, dementia, organic mental disorder, pervasive developmental disorder, schizophrenia, psychotic disorder, or bipolar disorder). Informed consent was obtained from all participants, and the study was conducted in accordance with the Declaration of Helsinki. Ethical approval was obtained from the Medical Ethics Committee (08.10.2025; Decision No. 2025/12 − 01).

## Data collection instruments

### Sociodemographic and clinical data form

This investigator-designed form included questions on age, sex, age at first smartphone ownership, estimated smartphone use duration, and the applications on which participants spent the most time.

### Smartphone addiction scale-short version

The SAS-SV is a 10-item, 6-point Likert-type self-report scale developed by Kwon et al. [12]. Higher scores indicate increased risk of smartphone addiction. The proposed cutoff values are ≥ 31 for men and ≥ 33 for women [12]. The Turkish validity and reliability study was conducted by Noyan et al. [13] .

### Mindful attention awareness scale

The MAAS is a 15-item, 6-point Likert-type self-report scale developed by Brown and Ryan [14]. It assesses the ability to attend to the present moment and maintain awareness in daily life. The Turkish adaptation was performed by Özyeşil et al. [15].

### Insight scale

The Insight Scale is a 20-item, 5-point Likert-type self-report instrument developed by Akdoğan et al. The internal consistency of the total scale was good, with a Cronbach’s alpha coefficient of 0.84 [16]. It evaluates cognitive awareness of one’s own psychological processes and the capacity for self-understanding. The scale includes the subdimensions of holistic perspective, self-acceptance, and self-understanding. Items 4, 9, 13, and 17 are reverse scored, and higher scores indicate greater insight in the relevant domain.

### Screen time

Participants were asked to report their average daily screen time by accessing the settings/weekly report/screen time section on their smartphones. Based on literature indicating increased mental and physical health problems among individuals with screen time ≥ 6 h per day, this threshold was used in our study to define intensive use [17].

### Operational definitions

In this study, intensive use was defined as daily screen time of 6 h or more, and problematic use was defined as an SAS-SV score at or above the sex-specific recommended cutoff value. Risky use was conceptualized as a composite variable created for the purposes of this study and was defined as meeting at least one of the two criteria for intensive use and problematic use (i.e., daily screen time ≥ 6 h and/or an SAS-SV score above the cutoff value).

## Statistical analysis

All statistical analyses were performed using SPSS software (version 24.0). The normality of variable distributions was assessed using both visual (histograms and Q-Q plots) and analytical (Kolmogorov-Smirnov/Shapiro-Wilk) methods. Descriptive statistics were presented as means for normally distributed variables and medians for non-normally distributed variables. Binary groups were formed according to average daily smartphone screen time (< 6 h and ≥ 6 h) and SAS-SV cutoff values (< cutoff and ≥cutoff). Between-group comparisons were performed using the chi-square test, Fisher’s exact test, Student’s t test, and Mann-Whitney U test, as appropriate. Insight and MAAS scores across groups were visualized with boxplots. Binary logistic regression analysis was performed to identify predictors of belonging to the risky use group. A type I error level below 5% was considered statistically significant.

## Results

A total of 297 university students were included in the study, comprising 180 women and 117 men. The mean age of the participants was 23.58 ± 5.04 years. The most common age at first mobile phone ownership was 11–15 years (60.6%), followed by 16 years or older (33.0%) and younger than 10 years (6.4%). The most frequently used applications were photo-sharing applications (33.0%), messaging/communication applications (27.6%), and video-sharing applications (20.5%). The distribution of participants according to daily screen time was as follows: 37.7% reported 4–6 h, 22.6% reported 2–4 h, 22.6% reported 6–8 h, 8.1% reported 8–10 h, and 7.1% reported 0–2 h.

When participants were compared according to daily screen time (< 6 h vs. ≥ 6 h), no statistically significant between-group differences were found in sex distribution, field of study, living arrangement, age at first smartphone ownership, or accuracy of estimating screen time (all *p* > 0.05). In contrast, a significant difference was observed in the type of platform most frequently used (χ² = 12.232, df = 4, *p* = 0.016): photo-sharing applications were more prevalent in the < 6-hour group, whereas video-sharing applications were more common in the ≥ 6-hour group (Table 1).

**Table 1:** Comparison of sociodemographic characteristics between participants with screen time below and above 6 hours

	Variable	Screen time <6 hour (*n*/%)	Screen time ³6 hour (*n*/%)	Statistical analysis*
Gender	Female	122 (61.0)	58 (59.8)	χ^2^ = 0.040
	Male	78 (39.0)	39 (40.2)	df = 1
				*p *= .842
Field of study	Arts and Design	5 (2.5)	2 (2.1)	
	Science and Literature	9 (4.5)	6 (6.2)	
	Dentistry	20 (10.0)	4 (4.1)	
	Educational Science	9 (4.5)	6 (6.2)	
	Engineering	13 (6.5)	7 (7.2)	χ^2^ = 8.709
	Law	12 (6.0)	4 (4.1)	df = 10
	Medicine	59 (29.5)	35 (36.1)	*p *= .560
	Natural Science	24 (12.0)	10 (10.3)	
	Political Science	10 (5.0)	1 (1.0)	
	Theology	11 (5.5)	5 (5.2)	
	Other	25 (12.5)	17 (17.5)	
Living arrangement	Alone	31 (15.5)	12 (12.4)	
	With roommate(s)	38 (19.0)	15 (15.4)	χ^2^ = 7.108
	In dormitory	41 (20.5)	30 (30.9)	df = 4
	With family	79 (39.5)	39 (40.2)	*p *= .130
	Other	11 (5.5)	1 (1.0)	
Most frequently used platform	Messaging and communication apps (WhatsApp, Telegram, BiP, etc.)	58 (29.0)	24 (24.7)	
	Photo-sharing apps (Instagram, Facebook, Snapchat, Pinterest, etc.)	70 (35.0)	28 (28.9)	
	Video-sharing apps (YouTube, TikTok, Vimeo, etc.)	31 (15.5)	30 (30.9)	χ^2^ = 12.232
				df = 4
	Social networking and discussion forums (X/Twitter, Reddit, etc.)	34 (17.0)	15 (15.5)	***p ***= .016^*^
	Others	7 (3.5)		
First phone age	5-10 Age	15 (7.5)	4 (4.1)	χ^2^ = 1.779
	11-15 Age	117 (58.5)	63 (64.9)	df = 2
	>16 Age	68 (34.0)	30 (30.9)	*p *= .407
Screen time accuracy	Accurate	146 (73.0)	65 (67.0)	χ^2^ = 1.139
	Inaccurate	54 (27.0)	32 (33.0)	df = 1
				*p *= .286

*p < .05, **p < .001, χ^2^ Chi-square test value, *df* degrees of freedom

When the groups formed according to SAS-SV scores (below cutoff vs. at or above cutoff) were compared, no statistically significant differences were found with respect to sex, field of study, age at first smartphone ownership, or accurate estimation of screen time (all *p* > 0.05). However, living arrangement differed significantly between the groups (χ² = 11.694, df = 4, *p* = 0.020). The high SAS-SV group was characterized by a higher rate of dormitory living, whereas the low SAS-SV group more frequently lived with family. A marked difference was also observed in the most frequently used platform type (χ² = 25.661, df = 4, *p* < 0.001); messaging/communication applications were more frequently reported in the below-cutoff group, whereas photo- and video-sharing applications were more common in the at-or-above-cutoff group (Table 2).

**Table 2:** Comparison of the sociodemographic characteristics of participants with SAS-SV scores below and above the cut off

	Variable	SAS-SV< cut off (*n*/%)	SAS-SV³ cut off (*n*/%)	Statistical analysis*
Gender	Female	89 (65.0)	91 (56.9)	χ^2^ = 2.022
	Male	48 (35.0)	69 (43.1)	df = 1
				*p *= .155
Field of study	Arts and Design	5 (3.6)	2 (1.3)	
	Science and Literature	8 (5.8)	10 (6.3)	
	Dentistry	11 (8.0)	13 (8.1)	
	Educational Science	8 (5.8)	7 (4.4)	
	Engineering	8 (5.8)	12 (7.5)	χ^2^ = 10.781
	Law	9 (6.6)	7 (4.4)	df = 10
	Medicine	36 (26.3)	58 (36.3)	*p *= .375
	Natural Science	21 (15.3)	13 (8.1)	
	Political Science	7 (5.1)	4 (2.5)	
	Theology	6 (4.4)	10 (6.3)	
	Other	18 (13.1)	24 (15.0)	
Living arrangement	Alone	20 (14.6)	23 (14.4)	
	With roommate(s)	25 (18.2)	28 (17.5)	χ^2^ = 11.694
	In dormitory	21 (15.3)	50 (31.3)	df = 4
	With family	64 (46.7)	54 (33.8)	***p ***= .020^*^
	Other	7 (5.1)	5 (3.1)	
Most frequently used platform	Messaging and communication apps (WhatsApp, Telegram, BiP, etc.)	53 (38.7)	29 (18.1)	
	Photo-sharing apps (Instagram, Facebook, Snapchat, Pinterest, etc.)	35 (25.5)	63 (39.4)	
	Video-sharing apps (YouTube, TikTok, Vimeo, etc.)	19 (13.9)	42 (26.3)	χ^2^ = 25.661
				df = 4
	Social networking and discussion forums (X/Twitter, Reddit, etc.)	24 (17.5)	25 (15.6)	***p ***= .000^**^
	Others	6 (4.4)	1 (0.6)	
First phone age	5-10 Age	6 (4.4)	13 (8.1)	χ^2^=3.877
	11-15 Age	79 (57.7)	101 (63.1)	df=2
	>16 Age	52 (38.0)	46 (28.7)	*p *= .144
Screen time accuracy	Accurate	95 (69.3)	116 (72.5)	χ^2^ = 0.358
	Inaccurate	42 (30.7)	44 (27.5)	df = 1
				*p *= .550

*p < .05, **p <. 001, χ2 Chi-square test value, *df* degrees of freedom

No significant between-group difference was observed in age according to daily screen time (< 6 h vs. ≥ 6 h) (*p* = 0.072, *r* = 0.10). The total MAAS score, which reflects mindfulness, was higher in the < 6-hour group (50 [26–68] vs. 46 [21–71], *p* < 0.001, *r* = 0.23). Regarding the subdimensions of the Insight Scale, the < 6-hour group had higher scores for holistic perspective (*p* = 0.039, *r* = 0.12) and self-understanding (*p* = 0.026, *r* = 0.13), whereas the self-acceptance score was higher in the ≥ 6-hour group (20 [9–29] vs. 17 [9–26], *p* < 0.001, *r* = 0.27). By contrast, no significant between-group difference was found in total insight score (*p* = 0.785, *r* = 0.02) (Table 3).

**Table 3:** Comparison of age, MAAS and IS scores across screen time and SAS-SV cutoff groups

Variable	Screen time <6 h Median (IQR)	Screen time ≥ 6 h Median (IQR)	*p* value *r*	SAS-SV < cutoff Median (IQR)	SAS-SV ≥ cutoff Median (IQR)	*p* value *r*
Age	24 (18–36)	22 (18–35)	0.072 0.10	25 (18–36)	23 (18–35)	**0.002** ^*^ 0.18
MAAS	50 (26–68)	46 (21–71)	**< 0.001** ^**^ 0.23	53 (21–71)	45 (26–70)	**< 0.001** ^**^ 0.44
Holistic perspective	16 (7–24)	15 (7–25)	**0.039** ^*^ 0.12	15 (7–24)	16 (7–25)	0.125 0.09
Self-acceptance	17 (9–26)	20 (9–29)	**< 0.001** ^**^ 0.27	17 (9–28)	19 (9–29)	**< 0.001** ^**^ 0.32
Self-understanding	13 (6–19)	12 (6–22)	**0.026** ^*^ 0.13	13 (6–21)	13 (6–22)	0.253 0.07
IS total	46 (29–62)	47 (29–70)	0.785 0.02	45 (29–67)	48 (29–70)	**< 0.001** ^**^ 0.22

*MAAS* Mindful Attention Awareness Scale, *IS* Insight Scale, *SAS-SV* Smartphone Addiction Scale-Short Version

*p < 0.05, **p< 0.001, values are presented as median (IQR). Comparisons were performed using Mann–Whitney U test

In the comparison based on SAS-SV cutoff values, the > cutoff group was younger (23 [18–35] vs. 25 [18–36], *p* = 0.002, *r* = 0.18) and had a lower total MAAS score (45 [26–70] vs. 53 [21–71], *p* < 0.001, *r* = 0.44). In contrast, self-acceptance (19 [9–29] vs. 17 [9–28], *p* < 0.001, *r* = 0.32) and total insight scores (48 [29–70] vs. 45 [29–67], *p* < 0.001, *r* = 0.22) were significantly higher in the > cutoff group. No significant between-group differences were found in the holistic perspective (*p* = 0.125, *r* = 0.09) or self-understanding (*p* = 0.253, *r* = 0.07) subdimensions (Table 3).

**Fig. 1:**
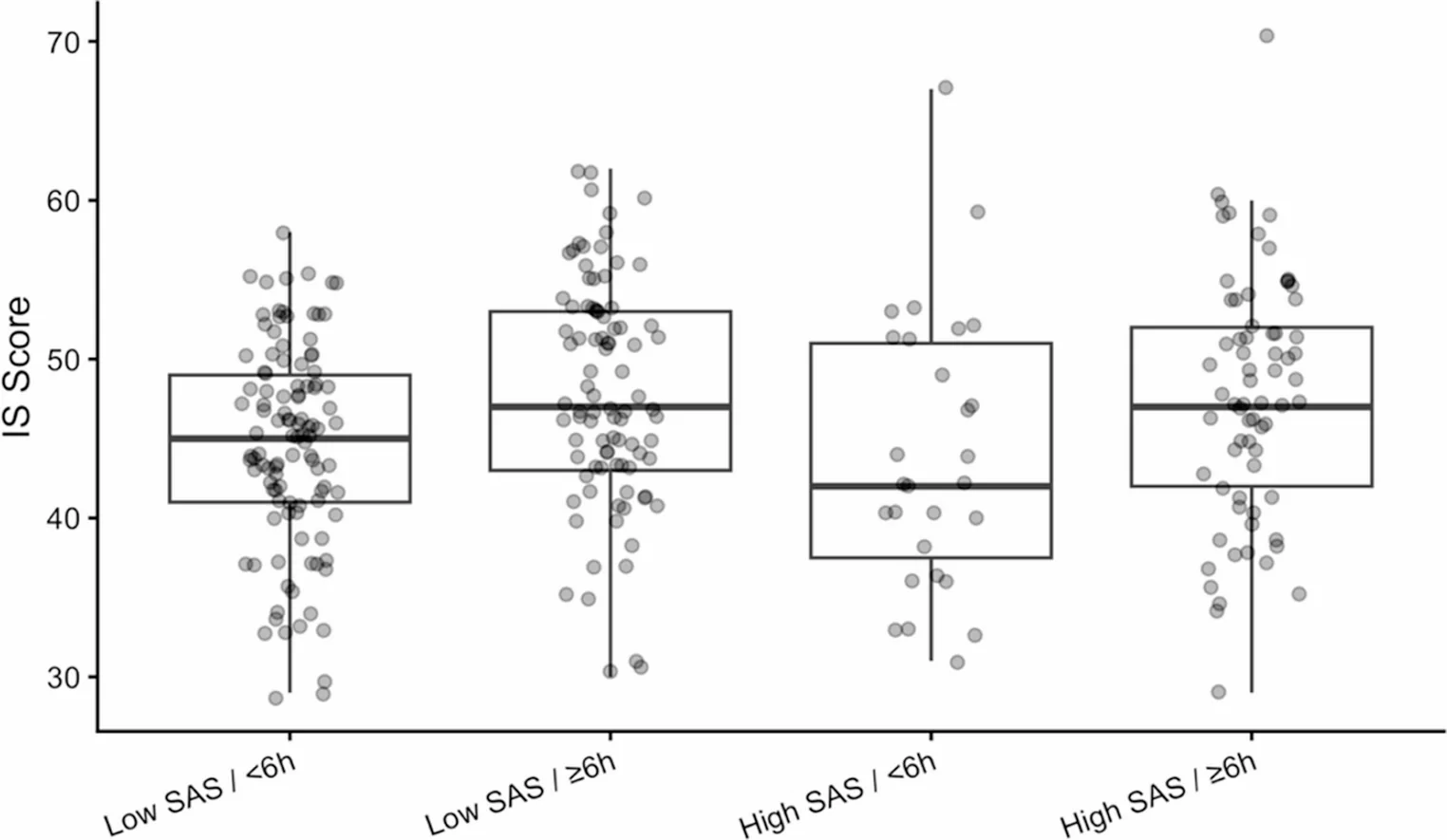
Comparison of insight scores across groups stratified by smartphone addiction risk and daily screen time Boxplot distribution of Insight Scale (IS) scores across four groups defined according to Smartphone Addiction Scale–Short Version (SAS-SV) status and daily smartphone use duration. Participants were categorized as having low SAS (below the sex-specific cut-off values) or high SAS (at or above the sex-specific cut-off values), and as having daily smartphone use of < 6 h or ≥ 6 h. Accordingly, four groups were formed: low SAS/<6 h, low SAS/≥6 h, high SAS/<6 h, and high SAS/≥6 h. The figure presents the distribution of IS scores in these subgroups

**Fig. 2:**
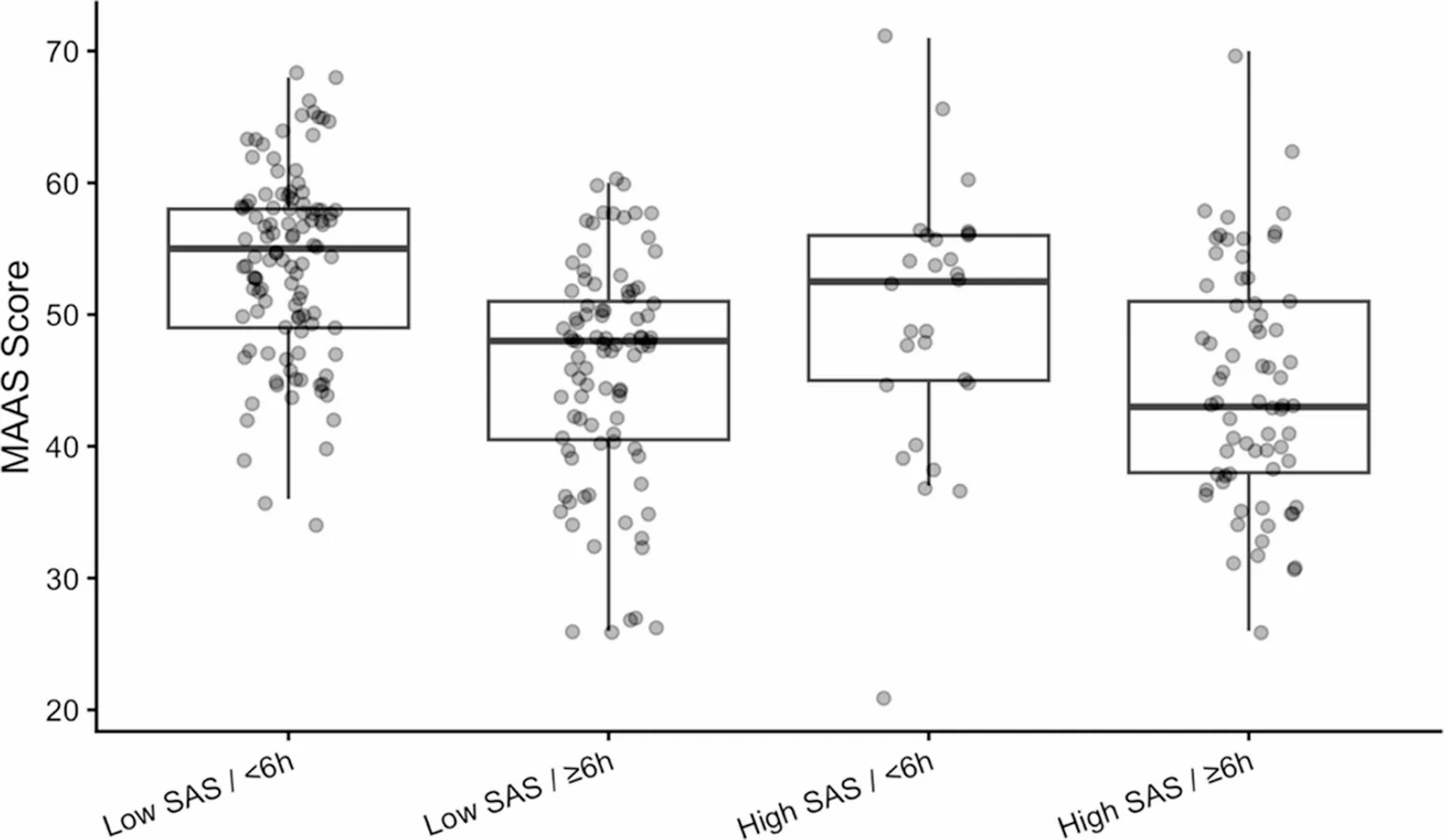
Distribution of MAAS scores across smartphone use profiles Boxplot distribution of Mindful Attention Awareness Scale (MAAS) scores across four groups defined by Smartphone Addiction Scale–Short Version (SAS-SV) status and daily smartphone use duration. Participants were classifie.d as low SAS or high SAS according to sex-specific SAS-SV cut-off values, and as having daily smartphone use of < 6 h or ≥ 6 h. Based on these criteria, four profile groups were formed: low SAS/<6 h, low SAS/≥6 h, high SAS/<6 h, and high SAS/≥6 h

Boxplots were used to examine the group-level distributions, median values, and spread of insight and mindfulness scores. The boxplot of insight scores showed that the group with high SAS-SV scores and < 6-hour of screen time had the lowest median insight score. However, the distribution within each group was wide, with several outliers above the upper limit. This finding suggests considerable individual variation (Fig. 1). In the boxplot of MAAS scores, the low SAS-SV/<6-hour group had the highest median MAAS score, while the high SAS-SV/≥6-hour group had the lowest median MAAS score (Fig. 2).

The Spearman correlation analysis indicated that higher SAS-SV scores were associated with lower MAAS levels (rs = -0.570, *p* < 0.01), higher self-acceptance scores (rs = 0.397, *p* < 0.01), and higher total insight scores (rs = 0.252, *p* < 0.01). MAAS showed a negative relationship with self-acceptance (rs = -0.462, *p* < 0.01) and overall insight (rs = -0.241, *p* < 0.01). Total insight showed strong to moderate positive links with the subdimensions of holistic perspective (rs = 0.822, *p* < 0.01), self-understanding (rs = 0.691, *p* < 0.01), and self-acceptance (rs = 0.541, *p* < 0.01). In addition, increasing age was associated with lower SAS-SV and self-acceptance scores, whereas MAAS scores increased slightly with age (Table 4).

**Table 4:** Spearman correlation matrix for age, SAS-SV, MAAS, and IS scores

	1	2	3	4	5	6	7
1. Age	1.000	− 0.188**	0.119*	0.011	− 0.203**	0.008	− 0.101
2. SAS-SV	− 0.188**	1.000	− 0.570**	0.086	0.397**	0.045	0.252**
3. MAAS	0.119*	− 0.570**	1.000	− 0.062	− 0.462**	0.057	− 0.241**
4. Holistic perspective	0.011	0.086	− 0.062	1.000	0.136*	0.627**	0.822**
5. Self-acceptance	− 0.203**	0.397**	− 0.462**	0.136*	1.000	− 0.050	0.541**
6. Self-understanding	0.008	0.045	0.057	0.627**	− 0.050	1.000	0.691**
7. IS total	− 0.101	0.252**	− 0.241**	0.822**	0.541**	0.691**	1.000

*MAAS* Mindful Attention Awareness Scale, *IS* Insight Scale, *SAS-SV* Smartphone Addiction Scale-Short Version

*p < 0.05, **p < 0.01 (two-tailed). Spearman’s rho coefficients are reported

Binary logistic regression analysis was performed to identify variables predicting membership in the risky use group. The model was significant (Omnibus χ² = 79.478, *p* < 0.001) and explained a moderate proportion of the variance in risky use classification (Cox & Snell R² = 0.235; Nagelkerke R² = 0.321). Overall classification accuracy was 74.1%, with a sensitivity of 86.2% and a specificity of 53.2%. In the multivariable model, age (B = -0.107, OR = 0.899, *p* < 0.001) and total MAAS score (B = -0.117, OR = 0.890, *p* < 0.001) independently and inversely predicted the likelihood of belonging to the risky use group; in other words, younger age and lower mindfulness were associated with a higher probability of risky smartphone use. By contrast, sex, most frequently used application type, total insight score, living arrangement, academic department, and age at first smartphone ownership were not identified as significant independent predictors in the model (all *p* > 0.05) (Table 5).

**Table 5:** Binary logistic regression model predicting membership in the risky smartphone use group

Variable	B	S.E.	Exp(B)	%95 CI	*p* value
Age	−0.107	0.033	0.899	0.842–0.959	**0.001** ^*^
Gender	0.336	0.294	1.400	0.786–2.490	0.252
Application type	−0.061	0.119	0.941	0.745–1.188	0.606
MAAS	−0.117	0.019	0.890	0.857–0.923	**< 0.001** ^**^
IS	0.013	0.020	1.014	0.974–1.054	0.504
Living arrangement	−0.178	0.130	0.837	0.649–1.080	0.170
Department	0.027	0.040	1.027	0.950–1.111	0.502
Age at first smartphone ownership	−0.186	0.274	0.831	0.485–1.421	0.498

*B* unstandardized regression coefficient, *SE* standard error, *OR* odds ratio, *MAAS* Mindful Attention Awareness Scale, *IS* Insight Scale, *SAS-SV* Smartphone Addiction Scale-Short Version

*p < 0.05, **p < 0.001

## Discussion

In this study, both time-based intensive smartphone use and self-report–based problematic smartphone use were evaluated together in university students, and an expanded risky-use phenotype encompassing both dimensions was defined. The main finding of the study was that risky smartphone use was particularly associated with younger age and lower levels of mindfulness. In contrast, although insight showed some associations in univariate analyses, it did not retain its status as an independent predictor in the multivariable model.

Although an association was found between time spent on smartphones and problematic use, these two concepts do not reflect the same construct. Daily screen time indicates the quantitative intensity of use, whereas scales such as the SAS-SV assess symptoms of problematic use, including loss of control, excessive mental preoccupation, and impairment in functioning. The literature likewise reports weak-to-moderate associations between screen time and problematic use. For example, problematic use has been reported to be more closely related to difficulties in emotion regulation, while objective screen time explains only a limited proportion of addiction scores [18, 19]. Therefore, it is not appropriate to classify every individual with high screen time as a problematic user. Similarly, some individuals with relatively low screen time may still exhibit marked symptoms of problematic use. Our findings support this distinction and suggest that approaches based solely on duration of use or scale scores alone may be insufficient for a more accurate understanding of smartphone use.

1. Mindfulness

In our study, mindfulness emerged as one of the strongest psychological correlates of risky smartphone use. Total MAAS scores were lower both in the high screen-time group and in the high SAS-SV group, and in the multivariable model MAAS remained a significant independent inverse predictor. This finding is consistent with previous studies reporting a negative association between mindfulness and problematic smartphone use. Meta-analytic evidence has shown a significant, moderate negative correlation between mindfulness and problematic smartphone use [10, 20]. This association has been reported to be stronger particularly in Eastern cultures and among women [10]. In contrast, the relationship between mindfulness and screen time appears to be weaker [11]. Our findings also support this distinction: whereas SAS-SV showed a moderate negative correlation with mindfulness (rs = -0.570, *p* < 0.001), the association between screen time and mindfulness was in the same direction but weaker (rs = -0.274, *p* < 0.001). These findings suggest that mindfulness may be more closely related to the problematic and psychologically dysregulated dimension of smartphone use than to the sheer quantity of use.

2. The Insight Factor

In our study, SAS-SV scores were positively correlated with self-acceptance (rs = 0.397, *p* < 0.001) and total insight (rs = 0.252, *p* < 0.001). At first glance, this could be interpreted as indicating that higher insight is associated with greater problematic smartphone use. However, the Insight Scale used in this study reflects constructs such as openness to criticism, willingness to confront one’s own flaws, tolerance of negative self-characteristics, and taking responsibility for one’s behavior, rather than the nonjudgmental acceptance emphasized in the mindfulness literature. The negative correlations observed between MAAS and both self-acceptance (rs = -0.462, *p* < 0.001) and total insight (rs = -0.241, *p* < 0.001) further support this interpretation. Accordingly, individuals with higher insight may recognize problematic aspects of their behavior more clearly and may therefore report them more explicitly on self-report measures. The lack of independent significance of insight in the multivariable analysis suggests that insight may be related less to the direct etiology of problematic smartphone use and more to how individuals perceive and report their own behavior.

3. The Age Factor

In this study, being younger was found to be an independent risk factor for risky smartphone use.This finding is consistent with the developmental neuropsychiatric literature. The balance between impulse control and reward sensitivity may not be entirely developed yet in early adulthood, and people may still have trouble controlling their emotions [21–23]. Indeed, higher impulsivity and lower mindfulness have been reported to be associated with problematic smartphone use [24]. Studies examining age-related differences in mindfulness have yielded mixed results, with some supporting and some not supporting higher mindfulness at older ages [25, 26]. In our sample, there was a weak positive relationship between age and mindfulness (rs = 0.119, *p* = 0.041). This finding may imply a gradual increase in mindfulness with age; however, the effect size is small and its clinical significance is limited. While younger age and lower mindfulness emerged as two meaningful determinants of risky smartphone use, the mechanisms through which being younger increases risk—such as impulsivity, emotion-regulation difficulties, or reward sensitivity—remain unclear.

Another age-related analysis in our study involved the age at first smartphone ownership. No significant difference was found in age at first smartphone acquisition in comparisons based on either daily screen time or SAS-SV cutoff values (*p* = 0.407 and *p* = 0.144, respectively). Although some studies have suggested that earlier smartphone ownership may be associated with adverse mental health outcomes [27, 28], our data indicate that earlier smartphone ownership was not associated with a significant increase in risk for either prolonged use or problematic use. However, our study did not assess potential adverse outcomes such as depression, anxiety, sleep disorders, or musculoskeletal complaints; therefore, these findings do not contradict the existing literature. In addition, because age at first ownership was based on retrospective self-report, recall bias and related reliability limitations should be acknowledged. Prospective cohort studies are needed to clarify the long-term psychological and somatic effects of early smartphone ownership.

4. Platform Type

There is a growing view that problematic use should not be conceptualized solely at the level of the device itself, but rather within a platform-specific framework such as problematic WhatsApp, Facebook, or Instagram use [29]. It is also assumed that different applications contribute to problematic smartphone use to varying degrees [30]. Indeed, applications such as WhatsApp, Instagram, YouTube, TikTok, and Facebook differ substantially in terms of use purpose, style of social interaction, content structure, and reward-related features. In line with the literature, the most frequently used platforms in our study were photo-sharing apps and video-sharing apps [31]. Furthermore, a significant difference in the most frequently utilized application type was observed between the groups when considering daily usage duration (≥ 6 h) and SAS-SV cutoff values in bivariate comparisons. Video-sharing applications, including YouTube, TikTok, and Vimeo, were more commonly used by individuals who spent ≥ 6 h per day on their smartphones. On the other hand, the group with higher SAS-SV scores preferred photo-sharing apps like Instagram, Facebook, Snapchat, and Pinterest more often. This finding is consistent with previous studies suggesting a possible association between frequent Instagram usage and problematic smartphone behavior [32]. While platforms like YouTube, which typically feature longer video content, may contribute to increased overall usage duration, the high SAS-SV group, distinguished by addiction-like symptoms, demonstrated a greater preference for platforms like Instagram. These platforms, which have fast content flow and stronger social feedback systems, directly affect impulsivity and reward sensitivity. Consequently, these findings imply that distinct behavioral outcomes may be elicited by different platforms.

5. Living Arrangement

Living arrangement is another important social-contextual variable that may influence risky smartphone use. Studies in adolescents have shown that those not living with their parents exhibit higher levels of smartphone addiction [12, 33]. In our study, when participants were classified according to screen time (< 6 h vs. ≥ 6 h), no significant between-group difference was found in living arrangement (χ² = 7.108, df = 4, *p* = 0.130). However, in the high-addiction-score group, the rate of dormitory residence was clearly higher (31.3% vs. 15.3%), whereas the rate of living with family was lower (33.8% vs. 46.7%), and this between-group difference was statistically significant (χ² = 11.694, df = 4, *p* = 0.020). This finding suggests that living arrangement may be associated more with the problematic pattern of use than with screen time alone. Living apart from family—particularly in dormitory settings—may contribute to smartphone use as an emotion-regulation or avoidance-based coping strategy in the context of stressors such as loneliness, concerns about social acceptance, and adjustment difficulties. Therefore, living arrangement should be considered a social variable that may shape the psychological quality of smartphone use.

## Limitations

Several limitations of this study should be acknowledged. First, because of the cross-sectional design, causal inferences cannot be drawn. Second, the relatively limited sample size and the inclusion of only university students restrict the generalizability of the findings. Third, because data were collected through questionnaires, most responses were based on self-report, which may have introduced response bias and measurement error. In addition, although screen-time data were based on device-recorded information, they were accessed and reported by participants themselves and were not independently verified; therefore, they should be interpreted as participant-mediated rather than fully objective measurements. Finally, the definition of risky use was an expanded composite classification developed for the purposes of this study; although this approach may be clinically useful, it may also encompass heterogeneous subgroups.

## Conclusion

This study demonstrated that risky smartphone use among university students was associated with younger age and lower levels of mindfulness. This finding highlights the potential importance of mindfulness-based approaches in preventive and interventional programs targeting risky smartphone use. Insight, by contrast, appeared to function more as a secondary variable influencing self-report style and displayed a heterogeneous pattern across groups. Variables including living situation and platform choice show that risky smartphone use is linked not just to how long smartphones are used, but also to how, where, and why they are used.

Our findings collectively suggest that risky smartphone use is not a singular phenomenon; instead, it is a multidimensional concept influenced by psychological traits, developmental variables, social context, and platform choices. Consequently, assessing smartphone usage exclusively through screen time or self-reported addiction scales may inadequately reflect the comprehensive nature of this behavior. Future research may benefit from employing more complete models that concurrently examine the psychological quality, contextual factors, and platform-specific patterns of smartphone use, rather than solely depending on total screen time or addiction scale scores.
